# Conversion study of hepatocellular carcinoma using HAIC combined with lenvatinib and PD-1/L1 immunotherapy under the guidance of BCLC staging

**DOI:** 10.3389/fimmu.2025.1596864

**Published:** 2025-06-02

**Authors:** Weihao Zhang, Xiaohui Zhao, Wei Gao, Tongguo Si, Qiang Zou, Xueling Yang, Wenge Xing, Haipeng Yu

**Affiliations:** ^1^ Department of Interventional Therapy, Tianjin Medical University Cancer Institute & Hospital, National Clinical Research Center for Cancer, Tianjin, China; ^2^ Tianjin's Clinical Research Center for Cancer, Tianjin Medical University Cancer Institute & Hospital, Tianjin, China; ^3^ Key Laboratory of Cancer Prevention and Therapy, Tianjin Medical University Cancer Institute & Hospital, Tianjin, China

**Keywords:** unresectable hepatocellular carcinoma, hepatic arterial infusion chemotherapy, lenvatinib, immunotherapy, conversion

## Abstract

**Objective:**

This study aimed to assess the efficacy and safety of hepatic arterial infusion chemotherapy (HAIC) combined with lenvatinib and immunotherapy and explore its potential as a conversion treatment for unresectable hepatocellular carcinoma (uHCC).

**Methods:**

A retrospective analysis was performed on clinical data from patients with uHCC who underwent HAIC, lenvatinib, and PD-1/PD-L1 immunotherapy. Data were collected from our hospital between November 2018 and December 2022. Efficacy was assessed based on the modified Response Evaluation Criteria in Solid Tumors (mRECIST). The primary endpoints were overall survival (OS), progression-free survival (PFS), and conversion therapy rate. Additionally, survival status curves were plotted using the Kaplan-Meier method. Lastly, prognostic risk factors affecting conversion therapy and survival outcomes were evaluated using Logistic and Cox regression models.

**Results:**

As of December 2022, 318 patients were included, comprising 40 patients (12.6%) in BCLC stage A, 123 patients (38.7%) in BCLC stage B, and 155 patients (48.7%) in BCLC stage C. The overall objective response rate (ORR) was 47.1%, whilst the disease control rate (DCR) was 85.5%. Meanwhile, the median overall survival (mOS) for the entire cohort was 21.7 months (95% CI: 19.7-24.3), with a median progression-free survival (mPFS) of 11.4 months (95% CI: 9.4-13.4). A total of 110 patients (34.6%) underwent conversion surgery. Multivariate logistic regression analysis identified BCLC stage as the sole independent risk factor affecting eligibility for conversion therapy. Subgroup analysis revealed that BCLC-B stage patients who achieved successful conversion therapy demonstrated significantly superior outcomes compared to those who did not undergo successful conversion therapy (median OS: 29.3 months [95% CI: 24.3-NA] vs. 19.7 months [95% CI: 17.2-24.6], P = 0.0013). Multivariate regression analysis identified the BCLC stage, the presence of distant metastasis, and receipt of conversion therapy as independent prognostic factors influencing OS. Among the cohort, 169 (53.1%) experienced grade 3-4 adverse events (AEs), with the most commonly reported AEs being fatigue, fever, and pain.

**Conclusion:**

The combination of HAIC with lenvatinib and immunotherapy yielded a high ORR, improved the conversion therapy rate, and prolonged both OS and PFS in patients with uHCC while maintaining a favorable safety profile. BCLC stage was identified as an independent prognostic factor influencing the success of conversion therapy, with patients in stage B deriving significant survival benefits post-conversion.

## Introduction

As is well documented, HCC is the sixth most prevalent cancer globally and the third leading cause of cancer-related deaths. By 2025, global liver cancer incidence is projected to exceed one million cases, with China, a high-incidence region, accounting for 47% of these cases (1). The majority of HCC patients are diagnosed at intermediate to advanced stages, rendering them ineligible for curative surgery or ablation, with a median survival time of merely 1 to 1.5 years (2). In recent years, advances in non-surgical treatments have enabled more patients with advanced liver cancer to undergo downstaging through conversion therapy, leading to improved survival benefits (3, 4).

The first-line standard treatment for advanced-stage liver cancer has been upgraded to a combination of targeted and immunotherapy (5, 6). To date, multiple large-scale Phase III clinical studies have validated the efficacy and safety of immune-targeted combination therapies for the treatment of patients with advanced hepatocellular carcinoma (HCC), achieving ORR ranging between 21% to 32.8% and a median OS between 19.2 to 22.1 months, significantly surpassing the efficacy of targeted therapy or immunotherapy alone (7, 8).

Conversion therapy refers to the treatment strategy for patients with uHCC, including those with poor overall physical conditions unable to tolerate surgical trauma or with compromised liver function precluding surgical intervention. This approach also encompasses tumor-specific conversion, wherein therapy aims to reduce tumor burden or downgrade the disease stage to facilitate curative R0 resection (9, 10). While targeted and immunotherapy have been established to confer survival benefits for patients with uHCC, the local control rate of the tumor is equally important, leading to the emergence of a conversion strategy integrating local therapy and targeted immunotherapy (11–13).

HAIC is effective in decreasing the intrahepatic tumor burden, given that it allows for the targeted delivery of chemotherapy drugs to the arteries supplying the tumor. The FOLFOX-HAIC treatment regimen (oxaliplatin, leucovorin, and fluorouracil), in conjunction with targeted therapy and immunotherapy, has preliminarily shown higher tumor response rates, improved survival outcomes, and greater potential for conversion therapy in patients with intermediate to advanced HCC and has become a clinical research hotspot (14, 15).

Thus, this study aimed to evaluate the safety and efficacy of FOLFOX-HAIC combined with lenvatinib and immunotherapy in HCC patients, explore factors influencing efficacy and prognosis, and identify factors affecting conversion therapy.

## Materials and methods

### Patients

This study retrospectively analyzed data collected from patients with uHCC who underwent HAIC combined with targeted therapy and immunotherapy at our hospital between November 2018 and December 2022. All patients were diagnosed based on the *Chinese Guidelines for the Diagnosis and Treatment of Primary Liver Cancer (2019 Edition)* and confirmed as uHCC through multidisciplinary team (MDT) consultations. The inclusion criteria were as follows: 1. age between 20 and 75 years; 2. Eastern Cooperative Oncology Group Performance Status (ECOG PS) score of 0-2; 3. Child-Pugh class A or B liver function; 4. absence of other malignancies. The exclusion criteria included an expected survival time of less than 3 months, severe liver dysfunction, severe splenic hyperfunction, a history of gastrointestinal bleeding, prior systemic or local treatment, and a history of liver or kidney transplantation and heart failure.

### Treatment protocol

HAIC was performed by puncturing the femoral artery using the modified Seldinger technique. During the procedure, a 2.7F microcatheter was super-selectively placed into the tumor-feeding artery to administer the drugs. The FOLFOX regimen used was as follows: a 4-hour infusion of 85 mg/m^2^ oxaliplatin, a 2-hour infusion of 400 mg/m^2^ calcium folinate and a bolus injection of 400 mg/m^2^ fluorouracil, followed by a 23-hour infusion of 1200-mg/m^2^ fluorouracil on day 1 of treatment. Dosages were adjusted based on the Child-Pugh score and chemotherapy tolerance. HAIC was repeated every 3-4 weeks until disease progression, the occurrence of unacceptable toxicity, or a change in the treatment plan. Upon the occurrence of grade 3 or 4 adverse events, the dosage of oxaliplatin was adjusted to 65 mg/m², while the dose of 5-fluorouracil was modified to 300 mg for each bolus administration and 1000 mg for each cycle.

### Molecular targeted and immunotherapy

Prior to or following the initial HAIC session, patients were intravenously injected with anti-PD-1 antibodies every 3 weeks, including 200 mg of sintilimab, 200 mg of tislelizumab, 200 mg of camrelizumab, 240 mg of toripalimab, or 200 mg of pembrolizumab. For anti-angiogenic therapy, patients were administered 8 mg lenvatinib orally once daily. To avoid adverse events, lenvatinib were discontinued for 2 days before and after HAIC. If the immunotherapy injection coincided with HAIC, it was postponed until 2 days after the procedure. Grade 3-4 AE measures including immunotherapy discontinuation, hormone therapy initiation, and combined immunosuppressants if necessary.

### Endpoints and evaluation of treatment effectiveness

The primary endpoints of the study were OS, PFS, and conversion therapy rate. Secondary endpoints included objective response rate (ORR, comprising complete response [CR] and partial response [PR]), disease control rate (DCR, comprising ORR and stable disease [SD]), and the incidence of adverse events (AEs) defined according to the Common Terminology Criteria for Adverse Events (CTCAE) version 4.0. Imaging assessments were conducted using the mRECIST (16). Evaluations were performed by two associate chief physicians or higher, each with more than five years of experience in imaging diagnostics for malignant tumors. Disagreements were adjudicated by a senior chief physician. Treatment continuation was considered when PR or SD was achieved, and the quality of life of patients did not decline. Conversion therapy was considered when CR or near-CR(≥90% reduction in enhancement) was achieved, and the treatment plan was accordingly adjusted in cases of PD. Treatment was suspended if severe adverse reactions occurred.

### Conversion of MDT treatment recommendations

Our Multidisciplinary Team (MDT) consists of interventional radiologists, hepatobiliary surgeons, medical oncologists, and diagnostic radiologists, who collaboratively participate in the entire treatment process including conversion therapy regimens and surgical timing/indications. Conversion Criteria: Child-Pugh A/B, ECOG ≤2, and imaging meeting oncological standards, with resection requiring FLR ≥40% & ICG-R15 ≤20%, and ablation allowing ≤5 cm solitary tumor or ≤3 lesions (each ≤3 cm).

### Statistical analysis

Statistical analysis was performed using SPSS 20.0 software and R programming, using independent samples t-test, rank sum test, chi-square test, and Kaplan-Meier curve analysis. Univariate and multivariate Cox regression analyses were conducted to identify prognostic factors, with P < 0.05 considered statistically significant.

## Result

### Clinical characteristics of patients

As of December 2022, 536 patients with uHCC underwent HAIC combined with molecular targeted and immunotherapy at our center, among which 318 met the inclusion criteria. Among the patients, 12.6% were in the BCLC-A stage, 38.7% in the B stage, and 48.7% in the C stage. Chronic hepatitis B virus infection was the most common cause of uHCC (73.3%). There were 115 cases (36.1%) with a single lesion and 203 cases (63.9%) with multiple lesions. Tumor size was categorized as <5 cm (31.9%), 5-10 cm (42.4%), and ≥10 cm (25.7%). Tumor location was unilateral in 144 cases (45.3%) and bilateral sides in 174 cases (54.7%). Vascular invasion was observed in 59 cases (18.6%), including 50 cases (15.7%) with portal vein tumor thrombus, and distant metastasis was noted in 52 cases (16.4%) (**Table 1**).

**Table 1:** Baseline characteristics and tumor profiles of patients.

Characteristics	Classifications	Patients (n=318)
Age (years)	<60	162 (50.9%)
	≥60	156 (49.1%)
Gender	Male	217 (68.2%)
	Female	101 (31.8%)
HBV infection	Yes	232 (73.0%)
	No	86 (27.0%)
BCLC stage	A	40 (12.6%)
	B	123 (38.7%)
	C	155 (48.7%)
Child-Pugh score	5	113 (35.5%)
	6	86 (27.0%)
	7	119 (37.4%)
Distant metastasis	No	266 (83.6%)
	Yes	52 (16.4%)
Vascular invasion	No	259 (81.4%)
	Yes	59 (18.6%)
AFP (ng/ml)	<400	98 (30.8%)
	≥400	220 (69.2%)
Number of tumors	Single	115 (36.2%)
	Multiple	203 (63.8%)
Tumor diameter (mm)	<50	102 (32.1%)
	50-100	133 (41.8%)
	≥100	83 (26.1%)
TBIL (g/L)		27.86±10.19
ALB (μmol/L)		39.38±5.82
ALT (U/L)		43.47±5.77
AST (U/L)		34.23±14.66
PLT (10*^9^/L)		163.38±66.92

The most frequently used immunotherapy drug was sintilimab, used in 136 cases, followed by tislelizumab in 108 cases, camrelizumab in 45 cases, atezolizumab in 17 cases, and toripalimab in 12 cases.

### Short-term efficacy

Follow-up was conducted at the end of every alternative course of HAIC, with all 318 patients completing the follow-up and receiving the next course of HAIC combined therapy. The first evaluation results revealed CR in 20 cases (6.28%), PR in 89 cases (28.0%), SD in 141 cases (44.3%), and PD in 68 cases (21.4%), with an ORR of 34.3% and a DCR of 78.6%. Among them, 8 patients underwent surgical resection after MDT discussion, and all postoperative pathological results were CR. At the second evaluation, excluding the 8 patients who underwent conversion resection, 310 patients completed the follow-up, with CR achieved in 43 cases (13.9%), PR in 103 cases (33.2%), SD in 119 cases (38.4%), and PD in 45 cases (14.5%), with an ORR and DCR of 47.1% and 85.5%, respectively. After the second evaluation, 102 additional patients underwent conversion surgery treatment following MDT discussion, among which 12 underwent radical radiofrequency ablation therapy (**Table 2**).

**Table 2:** Tumor treatment response assessment.

Efficacy Evaluation	The initial efficacy assessment	Conversion rate	The second efficacy evaluation	Conversion rate
	(n=318,%)		(n=310,%)	
CR	20(6.28)	8(2.5) ^#^	43(13.9)	102(32.9)^#^ 12(3.9)*
PR	89(28.0)		103 (33.2)	
SD	141 (44.3)		119 (38.4)	
PD	68(21.4)		45 (14.5)	
ORR	109 (34.3)		146 (47.1)	
DCR	250 (78.6)		265(85.5)	

^#^Surgical operation, *radiofrequency ablation (RFA).

### Conversion therapy analysis

In the present study, 110 patients (34.6%) underwent conversion therapy. Among them, 8 cases (2.5%) met the criteria for conversion resection at the first evaluation, whereas 102 cases (32.9%) proceeded to conversion therapy after the second evaluation, including 12 cases of radical radiofrequency ablation. Among patients who underwent conversion therapy, the majority were classified as stage B (60 cases, 54.5%), followed by stages A and C, with 27 cases (24.5%) and 23 cases (20.9%), respectively. Among patients with vascular invasion, 12 cases (20.3%) successfully underwent conversion therapy, while those with distant metastasis did not undergo conversion therapy. Significant differences were noted between the conversion therapy group and the non-conversion therapy group in terms of BCLC staging, distant metastasis, vascular invasion, AST levels, and platelet counts. Multivariate analysis identified BCLC staging as the only independent risk factor affecting conversion therapy (**Tables 3**, **4**).

**Table 3:** Comparison of clinical data between the conversion therapy group and non-conversion therapy groups.

Characteristics	Classifications	Non-conversion group	Successful conversion group	P-vale
Patients	318	208	110	
Age(years)	<60	107 (51.4%)	55 (50.0%)	0.899
	≥60	101 (48.6%)	55 (50.0%)	
Gender	male	146 (70.2%)	71 (64.5%)	0.367
	female	62 (29.8%)	39 (35.5%)	
HBV infection	Yes	157 (75.5%)	75 (68.2%)	0.207
	No	51 (24.5%)	35 (31.8%)	
BCLC stage	A	13 (6.2%)	27 (24.5%)	<0.001
	B	63 (30.3%)	60 (54.5%)	
	C	132 (63.5%)	23 (20.9%)	
Distant metastasis	No	156 (75.0%)	110 (100.0%)	<0.001
	Yes	52 (25.0%)	0 (0.0%)	
Vascular invasion	No	161 (77.4%)	98 (89.1%)	0.016
	Yes	47 (22.6%)	12 (10.9%)	
AFP (ng/ml)	<400	70 (33.7%)	28 (25.5%)	0.168
	≥400	138 (66.3%)	82 (74.5%)	
Number of tumors	Single	76 (36.5%)	39 (35.5%)	0.945
	Multiple	132 (63.5%)	71 (64.5%)	
Tumor diameter(mm)	<50	69 (33.2%)	33 (30.0%)	0.841
	50-100	86 (41.3%)	47 (42.7%)	
	≥100	53 (25.5%)	30 (27.3%)	
Child-Pugh Score	5	76(36.5%)	37(33.6%)	0.498
	6	59(28.4%)	27(24.5%)	
	7	73(35.1%)	46(41.8%)	
TBIL(g/L)		28.50±10.40	26.64±9.72	0.122
ALB(μmol/L)		39.34±5.70	39.46±6.06	0.861
ALT(U/L)		43.28±15.67	43.85±16.03	0.761
AST(U/L)		32.82±14.54	36.91±14.58	0.018
PLT(10*^9^/L)		157.66±64.36	174.19±70.53	0.036

**Table 4:** Multivariate analysis of factors influencing conversion therapy.

Characteristics	Odds Ratio (OR)	Lower 95% CI	Upper 95% CI	p-value
BCLC	0.472	0.221	1.005	0.052
	0.121	0.047	0.310	<0.001
Metastasis*	0.000	0.000	0.000	0.997
PVTT	1.466	0.57	3.775	0.427
AST	1.011	0.993	1.029	0.241
PLT	1.003	1.001	1.007	0.083

* With distant metastasis, no successful conversion occurred; strong negative predictive effect with OR approaching zero.

### Prognostic analysis

**Figure 1:**
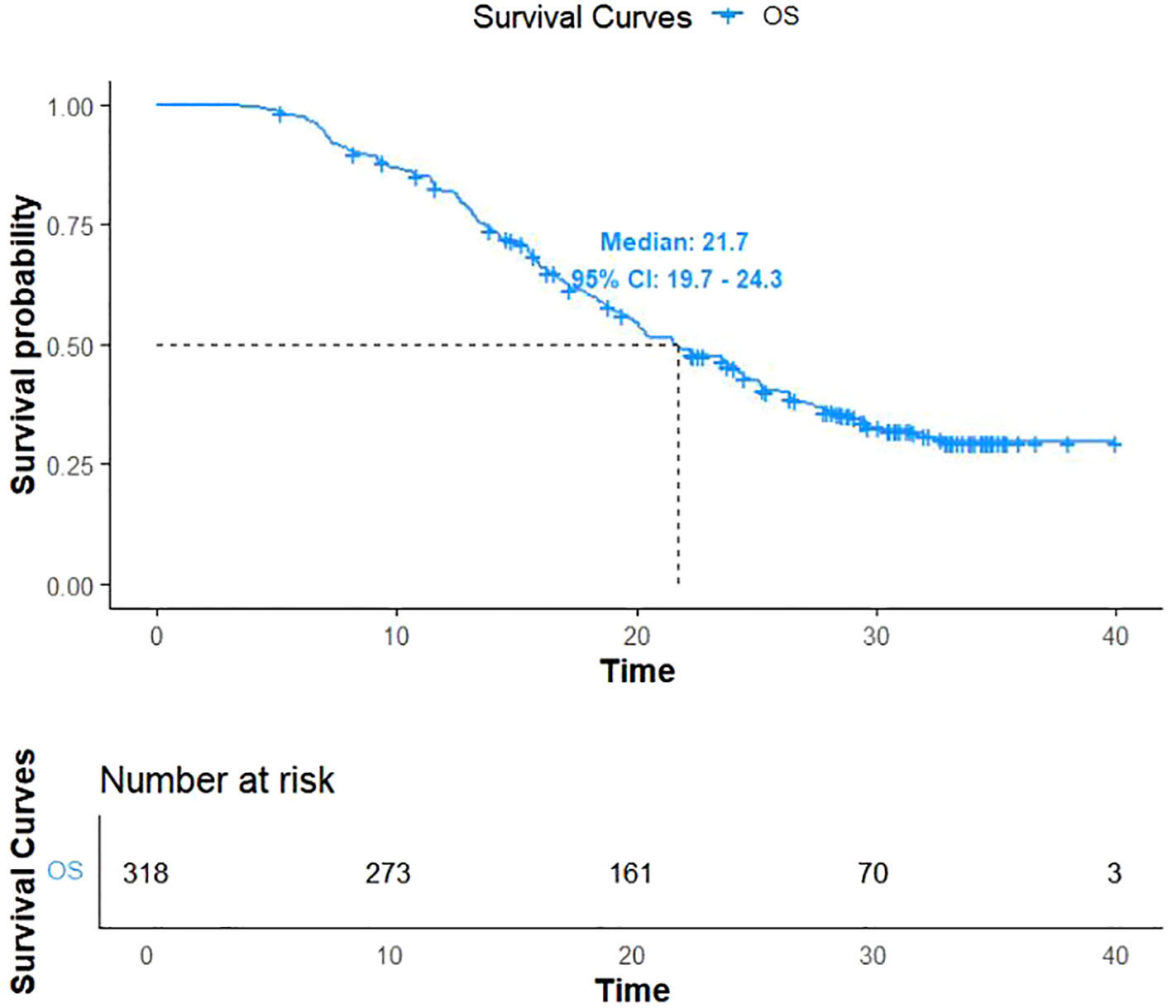
Kaplan–Meier curve for overall survival.

**Figure 2:**
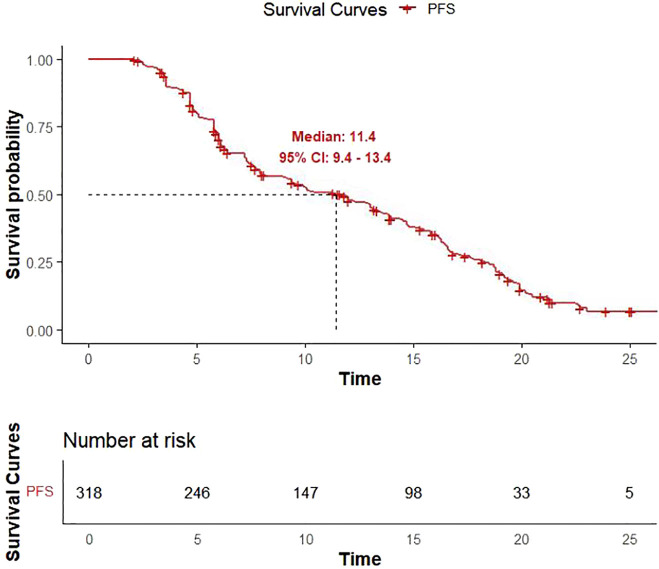
Kaplan-Meier curve for progression-free survival.

As of December 31, 2022, 206 patients (64.7%) reached the study endpoints, while the remaining 112 patients were either lost to follow-up or still alive at the time of follow-up cutoff. Kaplan-Meier curves for OS and PFS for all patients are illustrated in **Figures 1**, **2**, respectively. The mOS and mPFS were 21.7 months (95% CI: 19.7-24.3) and 11.4 months (95% CI: 9.4-13.4), respectively.

**Figure 3:**
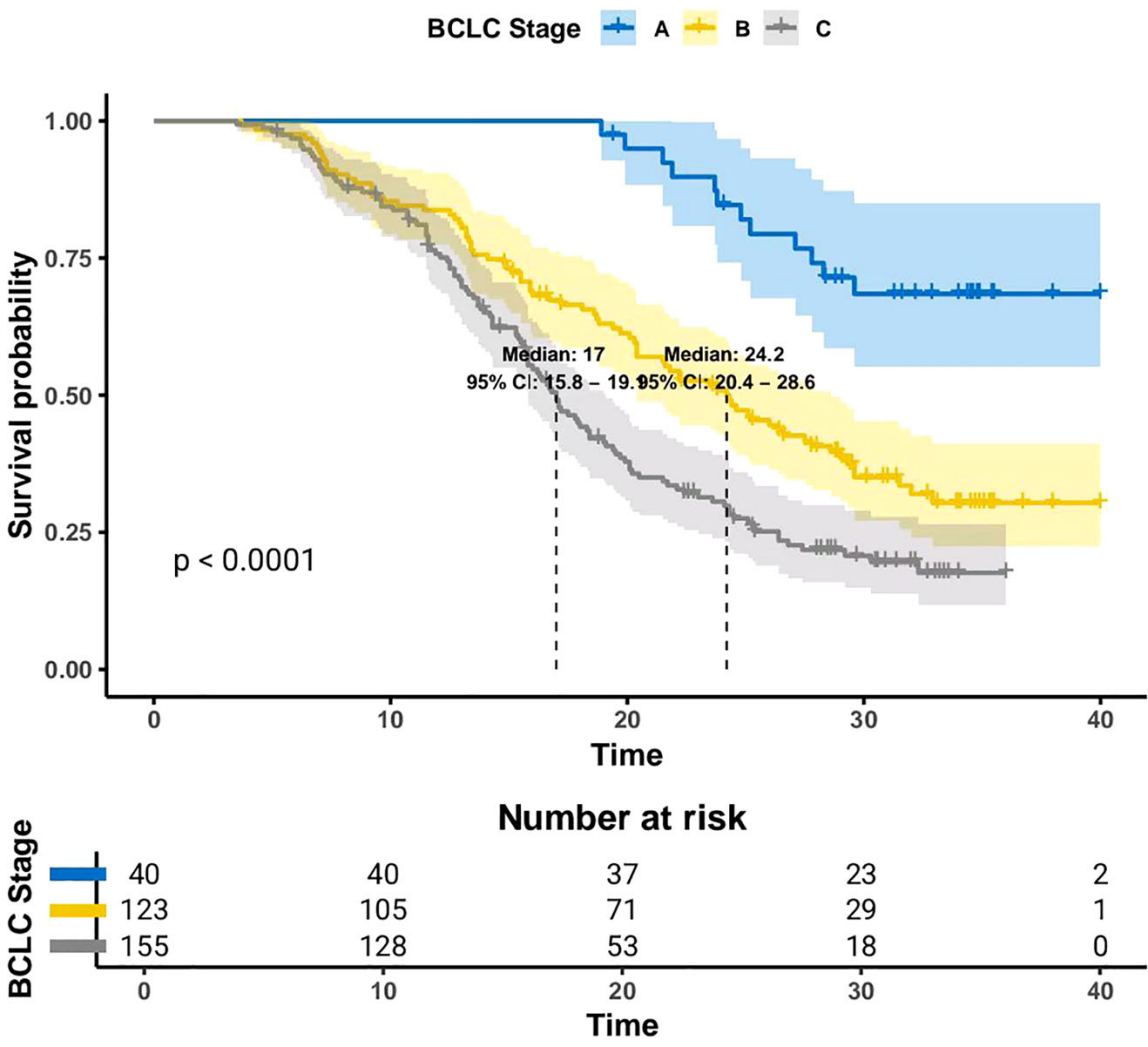
OS outcomes for patients with BCLC stages A, B, and C.

Furthermore, Kaplan-Meier survival curves were generated to assess mOS across different BCLC stages. Interestingly, patients with BCLC stage A had not reached mOS, with over 50% remaining alive at the end of follow-up. Regarding BCLC stage B and C patients, mOS was 24.2 months (95% CI: 20.4–28.65) and 17.0 months (95% CI: 15.8-19.1), respectively. As anticipated, survival times were significantly longer in stage B patients compared to stage C patients (P < 0.001) (**Figure 3**).

### Subgroup analysis

**Figure 4:**
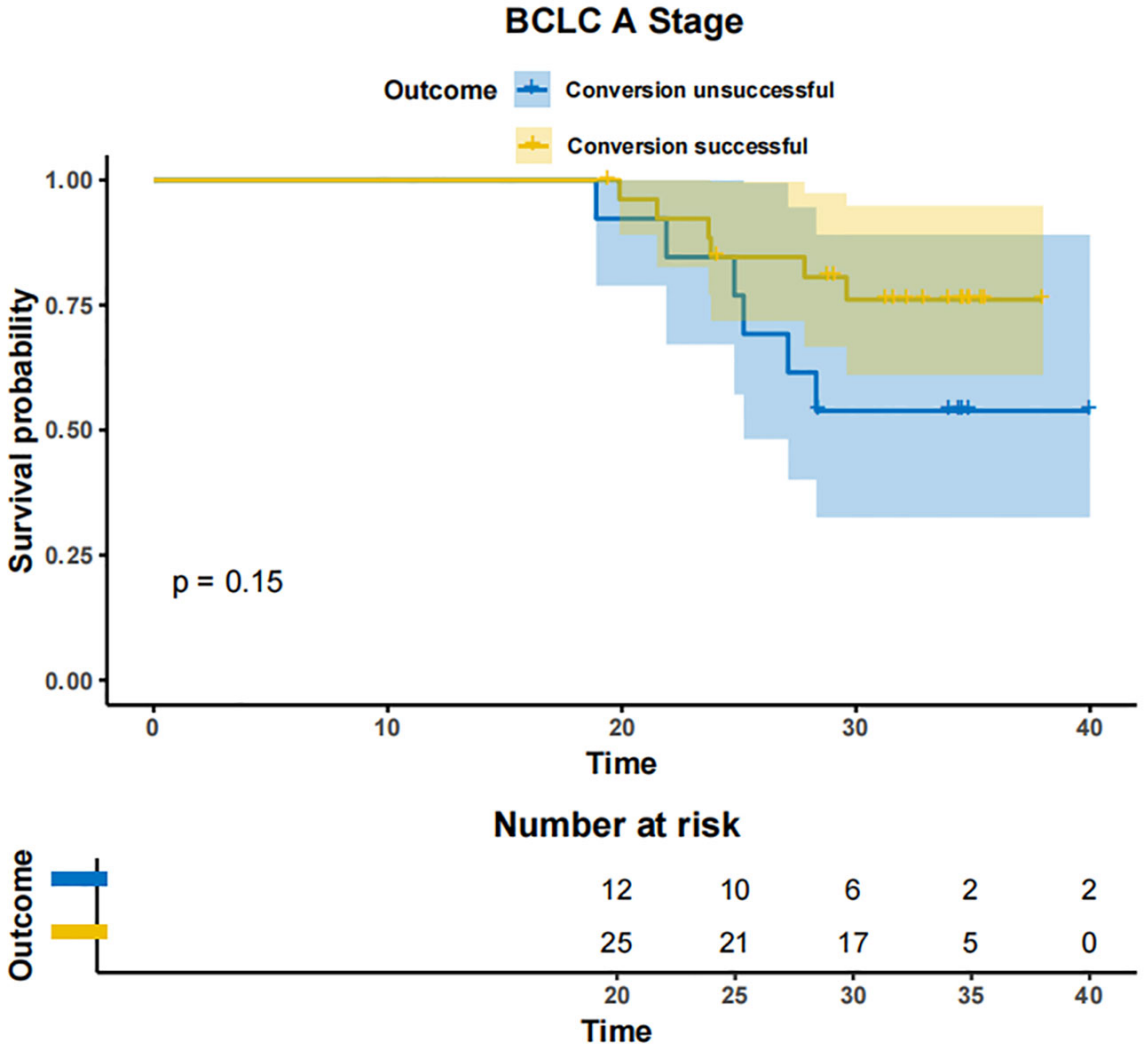
Conversion therapy and prognosis of patients with stage A BCLC.

**Figure 5:**
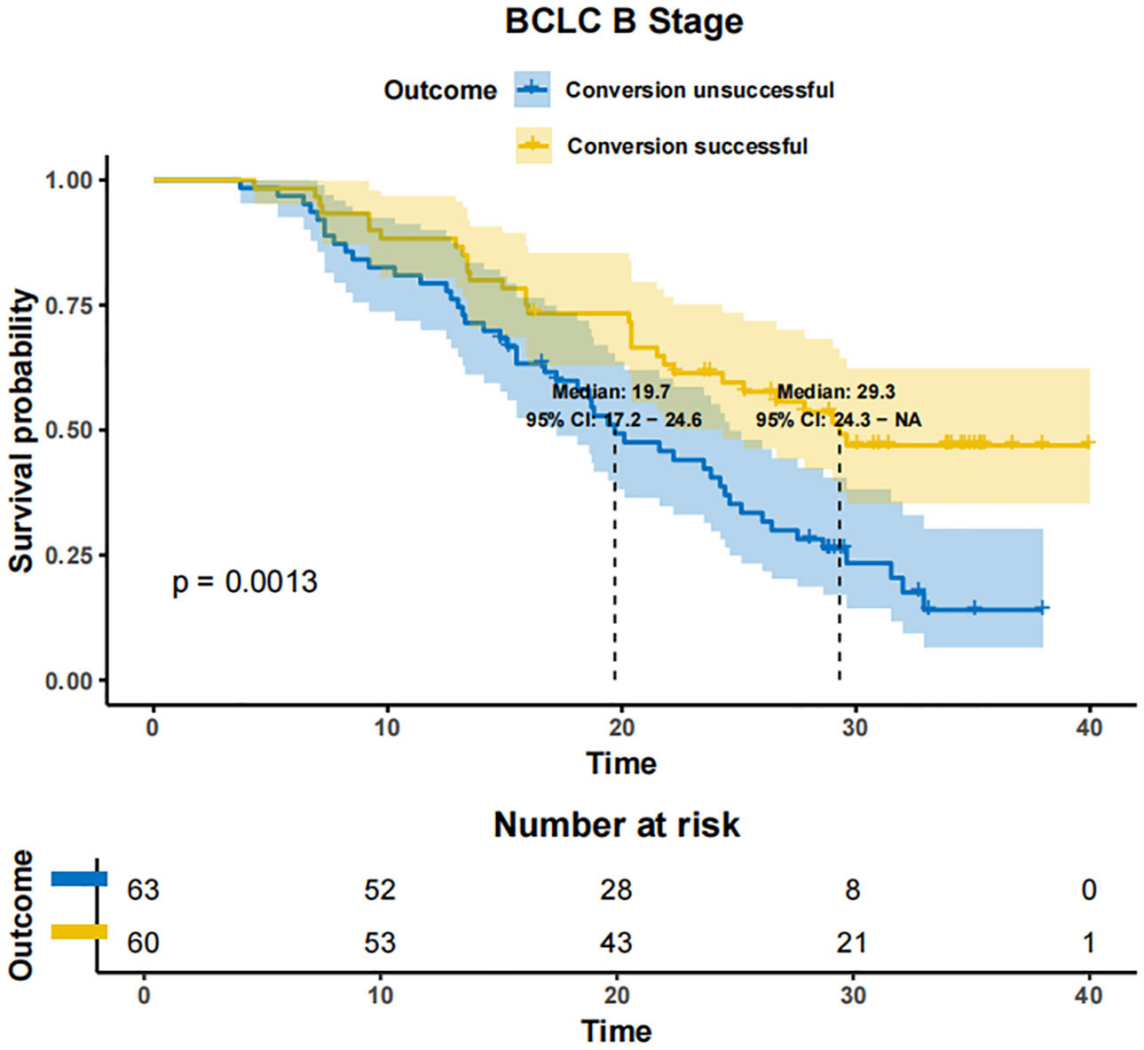
Conversion therapy and prognosis of patients with stage B BCLC.

**Figure 6:**
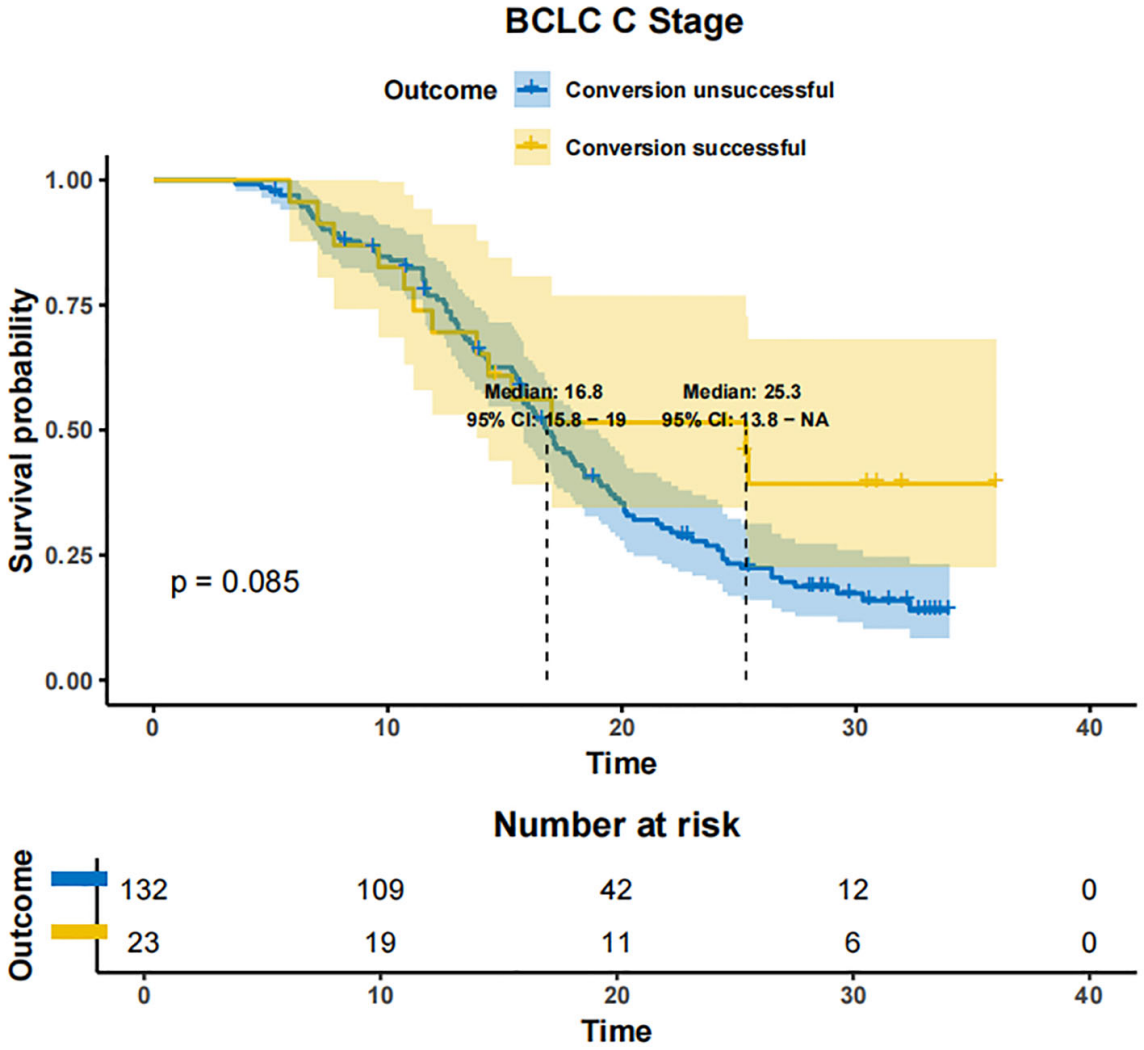
Conversion therapy and prognosis of patients with stage C BCLC.

In patients with BCLC-A stage disease, the mOS was not reached in both the successfully converted treatment and non-conversion therapy groups, and no statistically significant difference was noted between the two groups according to the KM curve trend (P=0.15) (**Figure 4**). In contrast, in BCLC-B stage patients, the mOS was 29.3 months (95% CI: 24.3-NA) in the conversion therapy group (60 cases) and 19.7 months (95% CI: 17.2-24.6) in the non-conversion group (63 cases), with a significant difference between the two groups (P=0.0013) (**Figure 5**). In BCLC-C stage patients, the mOS was 25.3 months (95% CI: 13.8-NA) in the conversion therapy group (23 cases) and 16.8 months (95% CI: 15.8-19.0) in the non-conversion group (132 cases), with no statistically significant difference between the two groups (P=0.085) (**Figure 6**).

**Figure 7:**
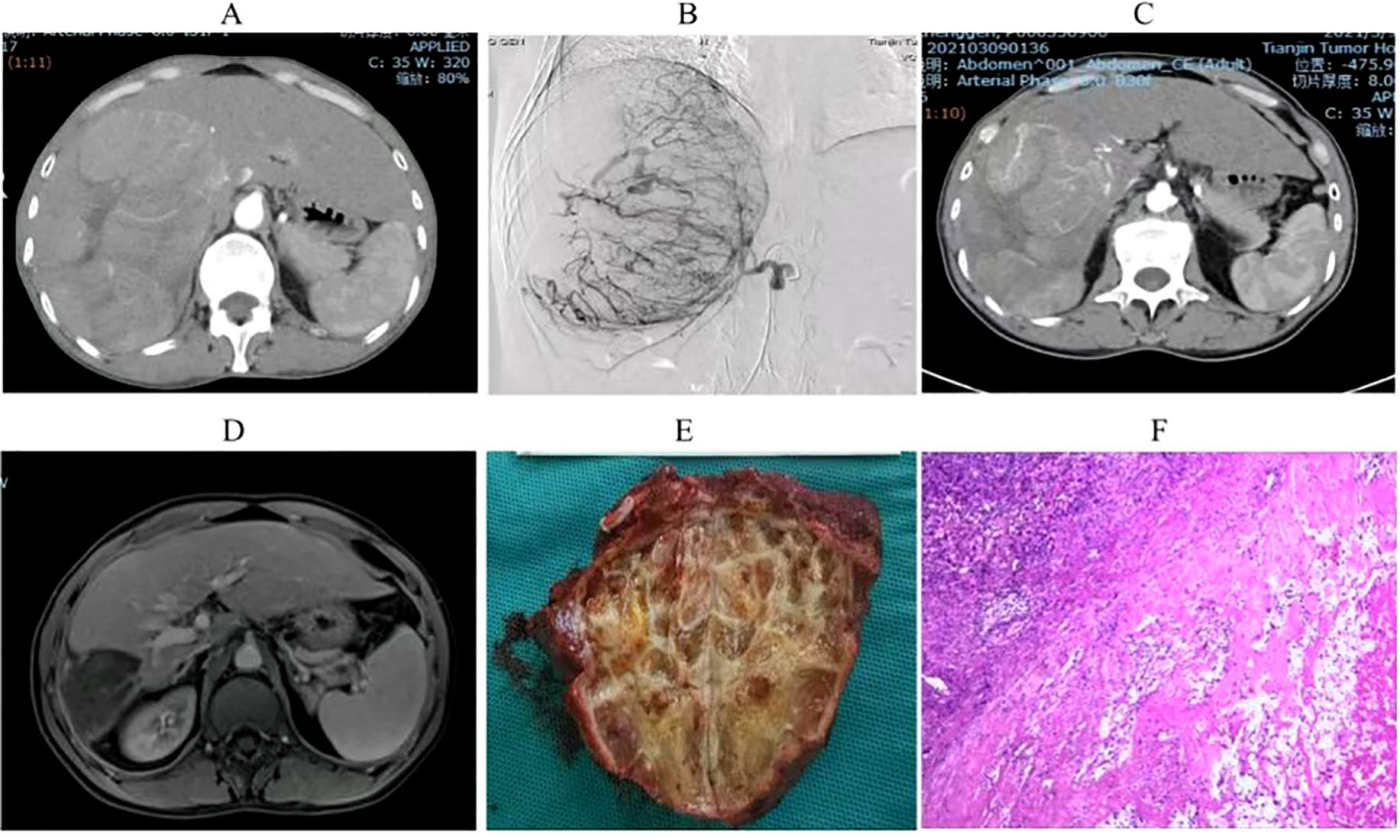
Imaging and pathological results of a uHCC patient who underwent conversion therapy following HAIC combined with lenvatinib and sintilimab. **(A)**. A large HCC measuring 15 cm in size was observed in the right lobe of the liver. **(B)**. Digital subtraction angiography (DSA) delineated a highly vascularized tumor. **(C)** Partial lesion shrinkage following two treatment cycles. **(D)** Significant reduction in tumor size after four treatment cycles. **(E)** Postoperative gross pathological specimens. **(F)**. Postoperative pathological examination showed complete tumor necrosis without residual tumor.

Univariate analysis identified four covariates associated with OS in patients, namely BCLC stage, the presence of distant metastasis, vascular invasion, and receipt of conversion therapy. These covariates were subsequently included in a multivariate Cox regression analysis using the direct entry method. The final results indicated that BCLC staging, presence of distant metastasis, and performance of conversion therapy are independent risk factors affecting OS (**Table 5**). A typical case is displayed in **Figure 7**.

**Table 5:** Univariate and multivariate prognostic analysis.

Variable	Univariate Analysis		Multivariate Analysis	
	Hazard Ratio (95% CI)	p-value	Hazard Ratio(95% CI)	p-value
Age	1.014 (0.770-1.330)	0.923		
Gender (male/female)	1.178 (0.880-1.570)	0.265		
HBV infection	1.150 (0.850-1.560)	0.373		
BCLC	3.281 (1.780-6.030)	<0.001	3.052 (5.246-276.246)	<0.001
	5.372 (2.950-9.770)	<0.001	3.157 (5.121-446.393)	<0.001
Metastasis	2.221 (1.560-3.160)	<0.001	1.524 (2.731-10.100)	0.048
Vascular invasion	1.744 (1.250-2.430)	0.001	1.371 (2.506-7.739)	0.123
AFP	1.289 (0.950-1.750)	0.103		
Tumor number (multiple/single)	1.101 (0.830-1.470)	0.513		
Tumor diameter	0.795 (0.580-1.090)	0.150		
	0.696 (0.480-1.000)	0.051		
Chlid-Pugh score	0.826 (0.587-1.163)	0.274		
	0.733 (0.531-1.010)	0.058		
TBIL	0.999 (0.99-1.010)	0.896		
ALB	0.99 0 (0.970-1.010)	0.428		
ALT	1.001 (0.990-1.010)	0.793		
AST	0.997 (0.990-1.010)	0.581		
PLT	0.999 (0.999-1.001)	0.614		
Successful conversion	0.393 (0.280-0.540)	<0.001	0.5197 (1.4412-2.0938)	<0.001

### Adverse events

During the follow-up period, 456 treatment-related AEs were reported. Among these, 287 were categorized as grades 1-2 and 169 as grades 3-4, with no fatal complications. Common grade 1-2 AEs included abdominal pain, fatigue, and hypertension, while grade 3-4 AEs comprised fatigue, fever, and pain. Sixteen patients experienced hypothyroidism, among which two cases were severe but resolved after treatment with levothyroxine. Meanwhile, twenty-five patients developed reactive cutaneous capillary endothelial proliferation (RCCEP), with four requiring corticosteroid therapy. Eleven patients developed subcutaneous hematomas and three experienced gastrointestinal bleeding; all were successfully treated. Additionally, three patients developed new-onset diabetes mellitus, which was adequately managed with insulin therapy (**Table 6**).

**Table 6:** Patient treatment-related adverse events.

Adverse Events	Grade 1-2(n,%)	Grade 3-4(n,%)
Abdominal pain	178 (39.06)	35 (7.7)
Fatigue	102 (22.4)	75 (16.4)
Vomiting	78 (17.1)	25 (5.5)
Diarrhea	16 (3.5)	5 (1.1)
Fever	95 (20.8)	30 (6.6)
Leukopenia	88 (19.3)	19 (4.2)
Thrombocytopenia	84 (18.4)	17 (3.7)
Pruritus	36 (7.9)	4 (0.7)
Hypertension	101 (22.1)	8 (1.9)
proteinuria	7 (1.5)	2 (0.4)
Hand-Foot syndrome	21 (4.6)	15 (3.2)
RCCEP	25 (5.5)	4 (0.9)
Immune-mediated pneumonia	10 (2.2)	2 (0.4)
Immune-related myocarditis	0 (0)	1 (0.2)
Hypothyroidism	18 (3.9)	2 (0.4)
Elevated ALT or AST	75 (16.4)	20 (4.4)
Subcutaneous hematoma	11 (2.4)	0 (0)
Gastrointestinal bleeding	3 (0.6)	0 (0)
Incident diabetes	3 (0.6)	0 (0)

## Discussion

Surgical resection remains the primary treatment for early-stage HCC patients. However, over 50% of patients are diagnosed at intermediate to advanced stages, rendering them ineligible for surgical modalities. For these patients, systemic therapy remains the primary treatment approach (17). The 2024 Clinical Practice Guideline for Primary Liver Cancer recommends the following first-line systemic therapies: atezolizumab combined with bevacizumab, sintilimab combined with a bevacizumab biosimilar, and spatinib mesylate combined with camrelizumab. Additionally, lenvatinib, donafenib, sorafenib, and the FOLFOX4 chemotherapy regimen are extensively employed as first-line treatments (18). Despite significant advancements in the treatment of advanced HCC in recent years, challenges persist. The limited benefits of systemic therapies, accompanied by challenges such as drug resistance and disease progression, remain major obstacles for the majority of advanced HCC patients (19, 20).

Conversion therapy regimens are recommended to adopt multi-combination approaches and high-ORR treatment strategies while ensuring safety (3, 21). Recent studies observed that for patients with uHCC, the combination of systemic therapy with local treatments such as transarterial chemoembolization (TACE) and HAIC could further improve the ORR and conversion therapy rate (22). Notably, according to earlier studies, approximately half of patients with intermediate-to-advanced HCC may achieve conversion to surgical resection opportunities through these approaches (23, 24).

HAIC was initially recommended by Japanese guidelines as the standard treatment for HCC with portal vein tumor thrombosis (25). At present, the combination of HAIC with immunotherapy and targeted drugs has shown significant efficacy (15, 26). A retrospective study undertaken by Professor Shi Ming from the Sun Yat-sen University Cancer Center demonstrated that compared to lenvatinib alone, the triple therapy of FOLFOX-HAIC combined with toripalimab and lenvatinib significantly extended the PFS and OS of patients with advanced HCC and concurrently improved the ORR. Of note, 14.1% of patients in the triple therapy group achieved CR (27). A randomized controlled trial enrolling HCC patients with portal vein tumor thrombus compared the efficacy of combination therapy with HAIC and sorafenib versus sorafenib monotherapy. The results demonstrated that the combination therapy group achieved a significantly higher overall response rate (40.8% vs. 2.46%) and longer PFS (7.03 months vs. 2.6 months). Additionally, 12.8% of patients in the combination therapy group experienced tumor downstaging and underwent R0 resection, with 3 patients achieving a pathological complete response after treatment (28). The TRIPLET phase II study assessed the efficacy and safety of camrelizumab, apatinib, and HAIC-FOLFOX in BCLC stage C HCC patients. The treatment achieved an ORR of 77.1% (RECIST v1.1) and a median PFS of 10.38 months. More importantly, 17.1% of patients experienced downstaging, enabling curative therapies, including R0 resection in five patients and curative ablation in one (29). Similar to previous studies, this real-world study demonstrated an ORR of 47.1% and a DCR of 85.5%, with 34.6% of patients undergoing radical surgical resection and ablation. The median PFS was 11.4 months, and the median OS was 21.7 months, significantly improving patient prognosis. We posit that the synergistic effect of interventional and targeted/immunotherapy may involve mechanisms such as the activation of various antitumor immune cells or the suppression of immune cells with tumor-promoting activity following interventional procedure (30). The discrepancies between the findings of this study and previous research may attributed to the use of lenvatinib monotherapy as the targeted agent in this study, with no restriction placed on the types of immunotherapeutic agents.

HAIC combined with systemic therapy demonstrates the ability to rapidly reduce tumor size and control intrahepatic lesions while exerting a relatively minor impact on liver function (31, 32). In certain cases, it may even enhance liver function reserve following a reduction in tumor burden (33). Furthermore, HAIC is associated with a diminished tissue inflammatory response and reduced adhesion post-surgery, thereby lowering surgical risks (34). Currently, several prospective clinical studies (NCT04961918, NCT05029973, NCT04947826, and NCT05003700) are underway to assess the efficacy of HAIC in conjunction with anti-PD-1/PD-L1 immunotherapy and molecular targeted therapy for advanced HCC, as well as the effectiveness of conversion therapy. We anticipate that their results will provide higher-level evidence-based medical evidence for the effectiveness of combined therapy (35).

Herein, among the 110 patients who underwent conversion therapy, the majority were in the BCLC-B stage. Further analysis unveiled that the mOS was 29.3 months in BCLC-B patients in the conversion group compared to 19.7 months in the non-conversion group, with a statistically significant difference. On the other hand, among BCLC-C patients, 23 underwent conversion surgery, and the remaining 132 patients did not undergo conversion surgery. While the mOS was 25.3 months in the conversion group and 16.8 months in the non-conversion group, the difference was not statistically significant, likely due to the limited sample size of converted patients in stage C. These findings collectively suggest that conversion therapy should be promptly initiated in BCLC-B patients if they meet the eligibility criteria (36). In comparison, conversion therapy should be approached with caution in BCLC-C patients, considering that the primary objective should extend beyond surgical resection to the development of individualized treatment strategies based on the patient’s overall condition.

In this study, almost all patients experienced at least one side effect, with 169 patients experiencing grade 3-4 AEs. Fatigue, fever, and pain were the most common grade 3-4 AEs. Three patients developed gastrointestinal bleeding, which was successfully managed with endoscopic hemostasis. The incidence of grade 3 and 4 AEs in this study was markedly higher than in previous studies, potentially due to the inclusion of adverse reactions associated with infusion chemotherapy (37, 38). Although these adverse reactions were classified as higher grades according to the CTCAE 4.0 criteria, they were largely manageable and minimally impacted patients’ overall prognosis and quality of life (39). Despite the high incidence of common AEs, they were generally effectively managed.

Nevertheless, some limitations of this retrospective study cannot be overlooked. To begin, the application of HAIC combined with immunotherapy and lenvatinib in clinical practice is relatively recent, resulting in limited overall follow-up time and insufficient clinical evidence for recommendations. Secondly, the retrospective design of this study inevitably introduced biases-even though we mitigated these through MDT-guided inclusion/exclusion criteria, independent dual data extraction with senior adjudication, multivariate regression adjustment for known prognostic factors, and BCLC-stratified subgroup analyses. Despite these limitations, HAIC-based combination therapy has demonstrated preliminary efficacy and favorable tolerability, showing promising prospects (40). Nonetheless, further prospective, multicenter clinical studies are warranted to identify patient populations that may derive benefits from this therapy, optimize treatment strategies, and refine treatment plans.

HAIC, combined with lenvatinib and immunotherapy, has demonstrated a high ORR in patients with uHCC. Besides, this combination reduced tumor burden, offered a favorable safety profile, increased the conversion therapy rate, and prolonged both OS and PFS. Finally, BCLC staging significantly impacted the likelihood of successful conversion therapy, with stage B patients deriving substantial survival benefits post-conversion.

## Data Availability

The original contributions presented in the study are included in the article/supplementary material. Further inquiries can be directed to the corresponding author.
